# DNA metabarcoding focusing on the plankton community: an effective approach to reconstruct the paleo-environment

**DOI:** 10.1038/s41598-023-48367-z

**Published:** 2023-12-08

**Authors:** Yasuhide Nakamura, Eri Ogiso-Tanaka, Koji Seto, Takuto Ando, Kota Katsuki, Yoshiki Saito

**Affiliations:** 1 Estuary Research Center, Shimane University, 1060 Nishikawatsu-Cho, Matsue, 690-8504 Japan; 2 Department of Botany, National Museum of Nature and Science, Tsukuba, 305–0005 Japan; 3 Center for Molecular Biodiversity Research, National Museum of Nature and Science, Tsukuba, 305–0005 Japan; 4 Graduate School of International Resource Sciences, Akita University, Akita, 010-0852 Japan

**Keywords:** Sedimentology, Limnology, Community ecology, Freshwater ecology, Ecosystem ecology, Marine biology

## Abstract

DNA metabarcoding (DNA-MB) targeting the whole plankton community is a promising approach in studies of sediment samples from water bodies, but its effectiveness in ancient material is not well demonstrated. We applied DNA-MB of plankton in a sediment core to reconstruct the paleo-environment of Lake Shinji, Japan, through a marine lagoon/freshwater lake transition during the past 2300 years. We interpreted core-sample plankton taxonomy and habitat by reference to the modern plankton community in water samples. OTUs (operational taxonomic units) belonging to Dictyochophyceae were 81.05% of the total reads in sediments. However, Ciliophora, Copepoda and Labyrinthulea formed the majority of plankton taxa in the water samples, suggesting that they are under-represented in sediment. A drastic change in plankton composition correlated with a large decrease in sediment sulfur concentration, implying the change of aquatic environment from marine lagoon to freshwater lake. This event took place ca. 1200 CE in Lake Shinji. A 250 year-long transitional period followed, during which the total DNA sequence reads were very low. This suggests that salinity fluctuations created a hostile environment for both marine and freshwater plankton species. Our results show that DNA-MB of the whole plankton community is effective in reconstructing paleo-environments.

## Introduction

Plankton are effective indicators of aquatic environments because of their basal trophic level, high abundance and biomass, and sensitivity to environmental changes due to their short life cycle. DNA metabarcoding (DNA-MB) is a widely used method of efficiently analyzing the plankton community, especially in field ecology studies^1^. This technique relies on sequencing bulk DNA from environmental samples to determine the taxonomic composition (species diversity) of the corresponding biota.

Analysis of sedimentary ancient DNA (sedaDNA) by DNA-MB has started to be used in the field of earth science, and many studies have focused on supergroup-level taxa or limited plankton groups (e.g., Dinoflagellata in Ref.^2^). DNA-MB targeting the whole plankton community is uncommon for several reasons. The great variety of planktonic organisms, belonging to various higher taxa, requires a very large knowledge base to interpret the output of DNA-MB. Reference DNA sequences registered in public databases (e.g., Genbank, SILVA) are useful for identifying taxa, but they sometimes contain sequences based on incorrect identifications. Ensuring a sound selection of data sources and correct identifications of taxa typically requires a team of taxonomic specialists.

Nonetheless, DNA-MB of the plankton community would be a very promising approach for paleo-environmental studies given the utility of plankton as ecological indicators. With this in mind, biologists and earth scientists collaborated in this study, in which the taxonomic affiliation and habitat of each taxon detected from an ancient context (a sediment core) were precisely identified by reference to DNA-MB data of the modern plankton community in water samples collected from the same lake system. To demonstrate the effectiveness of this method in studying ancient environmental change, a sediment core was obtained from a lake for which drastic ecosystem changes have been reported.

**Figure 1:**
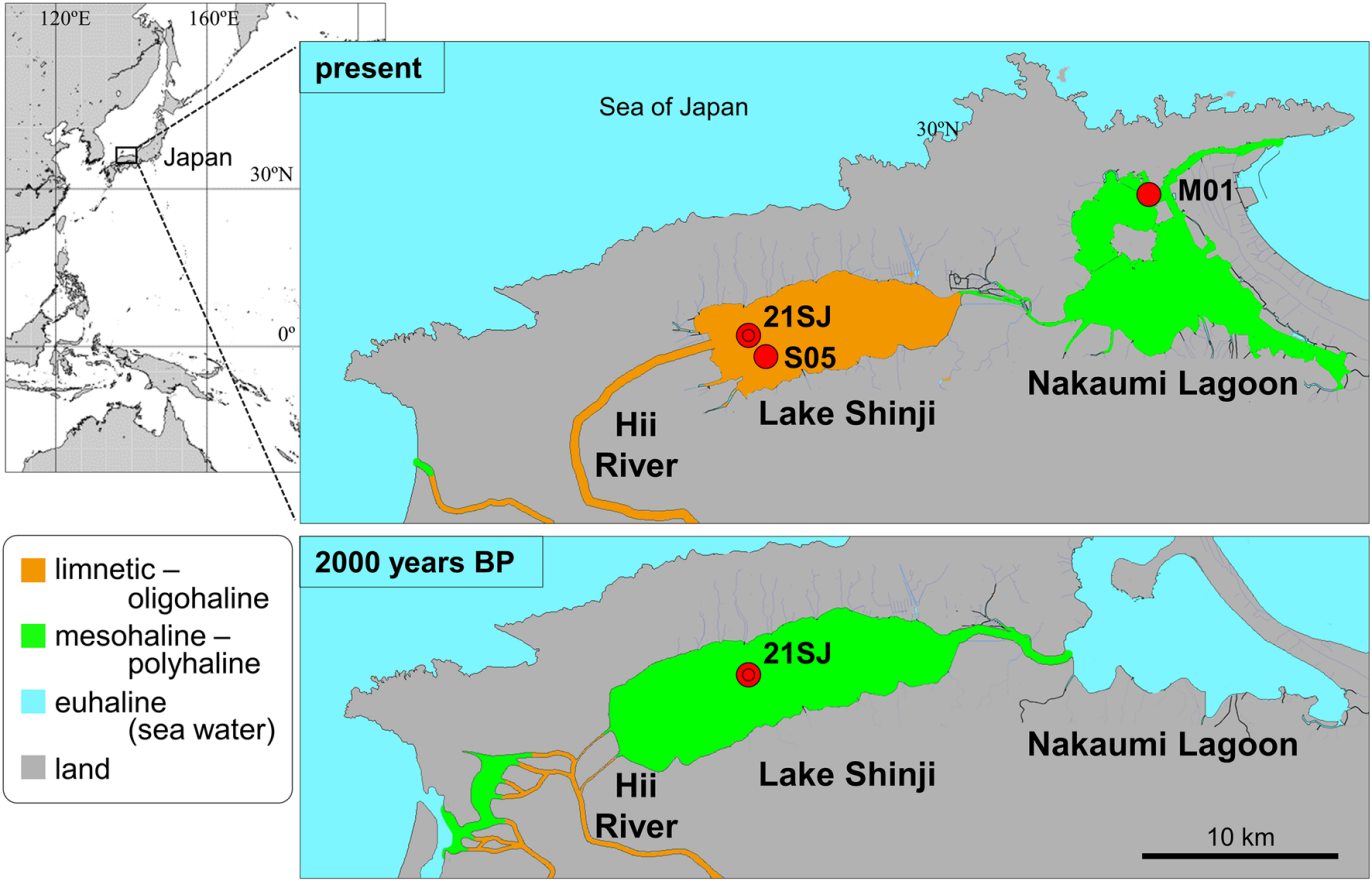
Locations of seasonal water samples (stations S05 and M01) and the sediment core (site 21SJ) in Lake Shinji and Nakaumi Lagoon. The map of 2000 years BP is based on Ref.^4^.

Lake Shinji, located in western Japan, (Fig. 1) currently has a low salinity (ca. 1–5), but it was a semi-closed bay 2,000 years ago^3, 4^. A large decrease in the sulfur concentration in sediment^5^ suggests that the bay became a freshwater lake during the past 2000 years, yet supporting biological evidence has been scant. Given this major environmental change and an anoxic environment in the lake floor that favors preservation of plankton DNA, this lake was chosen for an evaluation of DNA-MB targeting the plankton community. A sediment core from the lake floor yielded evidence that revealed the taxonomic and habitat composition of plankton over most of the time period represented by the core. To supplement previous studies of the plankton composition of the lake^6–8^, we also obtained reference data for living plankton taxa in the same lake and in an adjacent brackish water body, Nakaumi Lagoon.

## Materials and methods

### Water sampling

Water samples were collected with a Kitahara sampler (2 L) (Rigo, Japan) from May 2020 to February 2021, four times in each season, at station S05 in Lake Shinji and station M01 in Nakaumi Lagoon (Fig. 1) to obtain reference DNA data for the local plankton community. Plankton samples were also collected at these stations with a vertical plankton net (mouth opening 80 cm, mesh size 60 µm) to confirm the presence in situ of the taxa detected by DNA-MB. The plankton samples and some of the water samples were observed under stereo and inverted microscopes, and their major taxa were identified and photographed. DNA extraction of water samples was conducted in a strictly specialized and sterilized laboratory for modern environmental DNA studies using strict contamination prevention protocols. Samples (ca. 500 mL) were filtered through a 1.0 μm PTFE membrane filter (Thermo Fisher Scientific, U.S.A.) and the filters stored at − 20 °C. For each water sample, three filters were prepared. Three untreated filters were also analyzed as negative controls. DNA was extracted from the frozen filters according to the method described in Ref.^9^, and DNA-MB was conducted on the filtrate. The V9 hypervariable region of approximately 315 base pairs in 18S ribosomal RNA was amplified by polymerase chain reaction (PCR) following the procedure in Ref.^10^. The structure of fusion primers, the contents of the reaction mixture, the thermal cycling conditions and the purification method were the same as in Ref.^11^, and V9-specific sequences were based on Ref.^12^: 1389F and 1510R. PCR amplification of the negative controls confirmed the absence of contamination. The purified reaction mixture was adjusted to 4 pM before amplicon sequencing using MiSeq (Illumina, U.S.A.). One run of sequencing was performed with MiSeq Reagent kit v3 (600 cycles) (Illumina, U.S.A.) following the recommended protocol and default settings.

The obtained data were analyzed with Claident ver. 0.2.2019.05.10 software^13, 14^. Chimera sequences and low-quality sequences (average quality scores < 30) were excluded. The sequences were then clustered into operational taxonomic units (OTUs) using a minimum identification score of 0.97. The OTU compositions of each water sample are summarized in a table, which lists sequences longer than 200 mer with at least 200 reads. These steps removed 12.1–98.1% of the original sequence reads in the samples. OTUs were identified to the genus or species level using Genbank and assigned to phylum- or class-level taxa according to^15, 16^. Relative abundances were derived from the ratio of total sequence reads and the sequence reads of each higher taxon. This identification process was conducted also for three negative controls to confirm the absence of contamination. The raw sequence data were deposited in the DNA Data Bank of Japan database (accession number DRA017262).

### Sediment core sampling

A sediment core, 397 cm long, was collected in July 2021 at site 21SJ in Lake Shinji (35°26.5100N, 132°54.2795E, water depth 5.10 m) (Fig. 1) using a Mackereth-type core sampler^17^. The top 65 cm of the core was disturbed and not analyzed. Below 65 cm, the core was undisturbed, and a previous study^5^ indicated that it encompassed the ecosystem change we sought to capture. Immediately after sampling, the core was split in a laboratory. One half was cut into 1-cm sections and stored at − 20 °C for age estimation and chemical analysis. Subsamples for DNA-MB were taken from the other half at intervals of 6 cm, using spatulas sterilized by burning, after removing the outer surface of the sampling location using other sterilized spatulas. Subsamples were then preserved at − 20 °C for 3 days.

Organic materials for radiocarbon analysis (leaves and seeds of terrestrial plants) were picked from three core samples under a stereo microscope. Ages were estimated following the procedure in Ref.^18^. Total sulfur concentration was measured with an elemental analyzer (Flash EA 1112, Thermo Fisher Scientific, U.S.A.) following the protocol of Ref.^18^. A total of 52 sediment samples taken at 6-cm intervals were analyzed by DNA-MB. The DNA was extracted only one time from each core sample, since the amount of sample was limited. The extraction was conducted with the DNeasy PowerSoil Pro Kit (QIAGEN, Germany) at a laboratory that specializes in ancient environmental samples. This laboratory, in which modern samples were never used, is different from that used for the water samples and also uses strict sterilization protocols. The extracts from the core samples (including three negative controls) were processed in the same sequencing run as the water samples. The treatment of low-quality, infrequent or too short sequences removed 1.79–53.25% of the original sequence reads in the core samples. After taxonomic identification, we excluded sediment-dwelling taxa (e.g., Polychaeta, Nematoda and Fungi) and parasites (e.g., Apicomplexa) that could not be confidently assumed to be in situ. The OTUs were classified into four salinity-based habitats (Table S1): limnetic–oligohaline, mesohaline–polyhaline, euhaline (seawater) and others. Habitats were assigned on the basis of DNA-MB results from the water samples (Table S2) and previous studies^6–8^. Relative abundances were calculated for each higher taxon and habitat. Cluster analyses based on the composition of higher taxa and habitats were also conducted to visualize the differences among the core samples. The details concerning the analysis are shown in Fig. S4. For Dictyochophyceae, the most commonly detected higher taxon, we clarified the correspondence between lineages and habitats by creating a phylogenetic tree including OTUs from the water and core samples.

## Results

**Figure 2:**
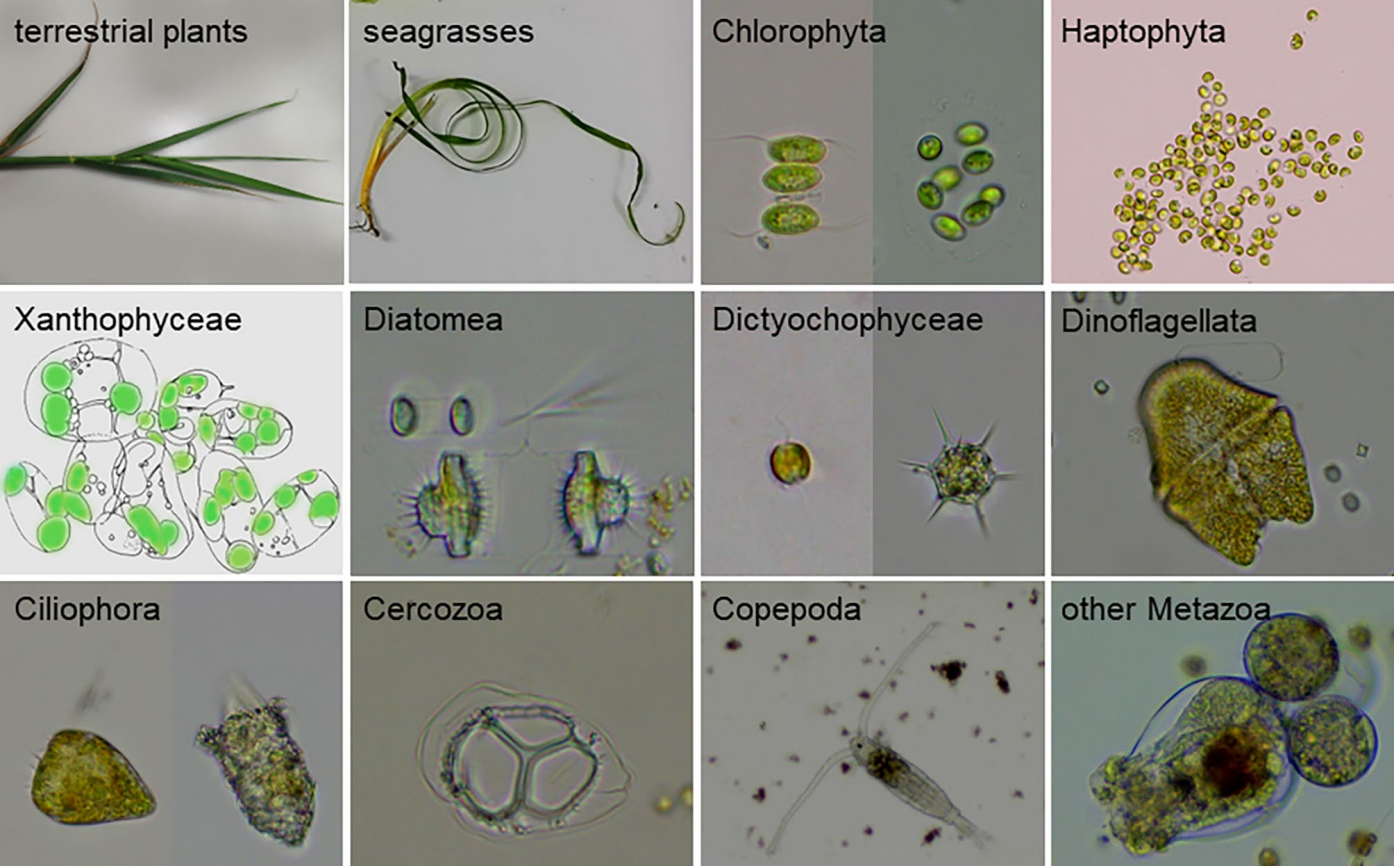
Examples of higher taxa detected in the core by DNA metabarcoding. Images show living specimens from Lake Shinji and Nakaumi Lagoon in 2020–2021 except Xanthophyceae, for which a schematic illustration is shown. The image of Haptophyta was provided by Dr. Hiroya Araie (Kanto Gakuin University).

**Figure 3:**
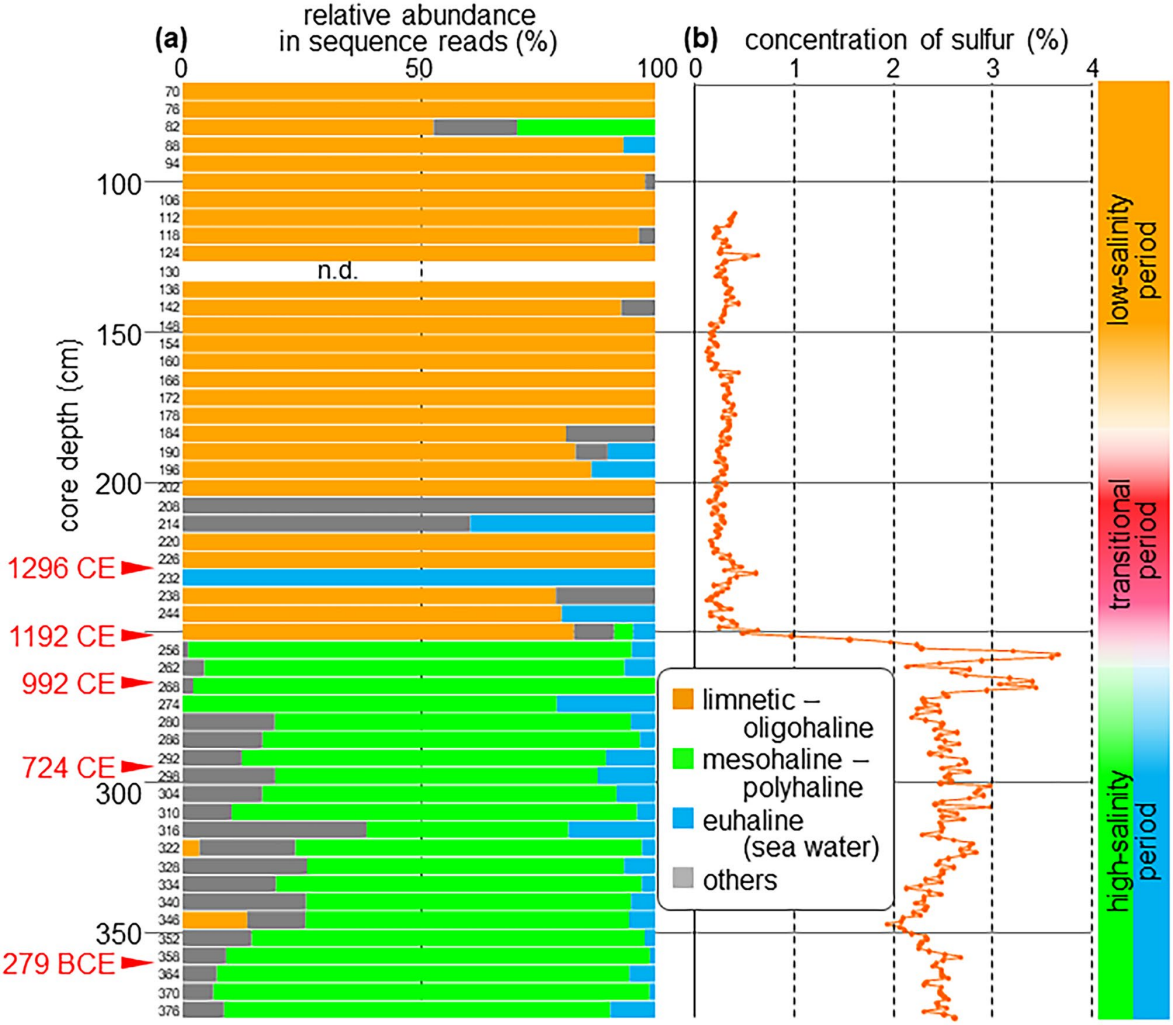
Vertical profiles of (**a**) composition of plankton habitats in sequence reads and (**b**) sulfur concentration in the core.

In the water samples, DNA-MB detected 154 OTUs from 21,101 sequence reads (Fig. S1). Total sequence reads ranged from 63 to 5,938 per sample (Table S2). The OTUs included about 50 genera and species belonging to 15 phyla or classes (Fig. 2, Fig. S1, Table S2). OTUs belonging to Ciliophora made up 29.91% of the total reads, followed by Copepoda (28.20%), Dinoflagellata (27.58%) and Labyrinthulea (2.75%). Habitats assigned based on the results from the water samples (Table S2) were then applied to the OTUs in the core samples (Fig. 3, Table S3).

We recognized three clades in the phylogenetic tree of Dictyochophyceae (Fig. S2). Given the paucity of information concerning Pedinellales (including *Pseudopedinella*), we reviewed previous studies and constructed a phylogenetic tree to assign habitats for this group. One clade (Pedinellales sp. 1) was classified as a freshwater lineage because we found three OTUs of this clade in modern freshwater. The other two clades (*Pseudopedinella* sp. 2 and *Pseudopedinella* sp. 3) were classified as brackish water lineages because the members of the genus are predominantly brackish water dwellers. The core consisted mainly of muddy sediment with almost no laminations. Radiocarbon analysis of plant tissues from the core yielded estimated calendar ages at five calibration points between 1296 CE and 279 BCE (Fig. 3, Table S4). Inferred sedimentation rates were higher (2.00–3.17 mm year^−1^) above 250 cm core depth than below it (0.64–1.08 mm year^−1^) (Fig. S3). Sulfur concentrations notably increased below about 250 cm (Fig. 3b).

**Figure 4:**
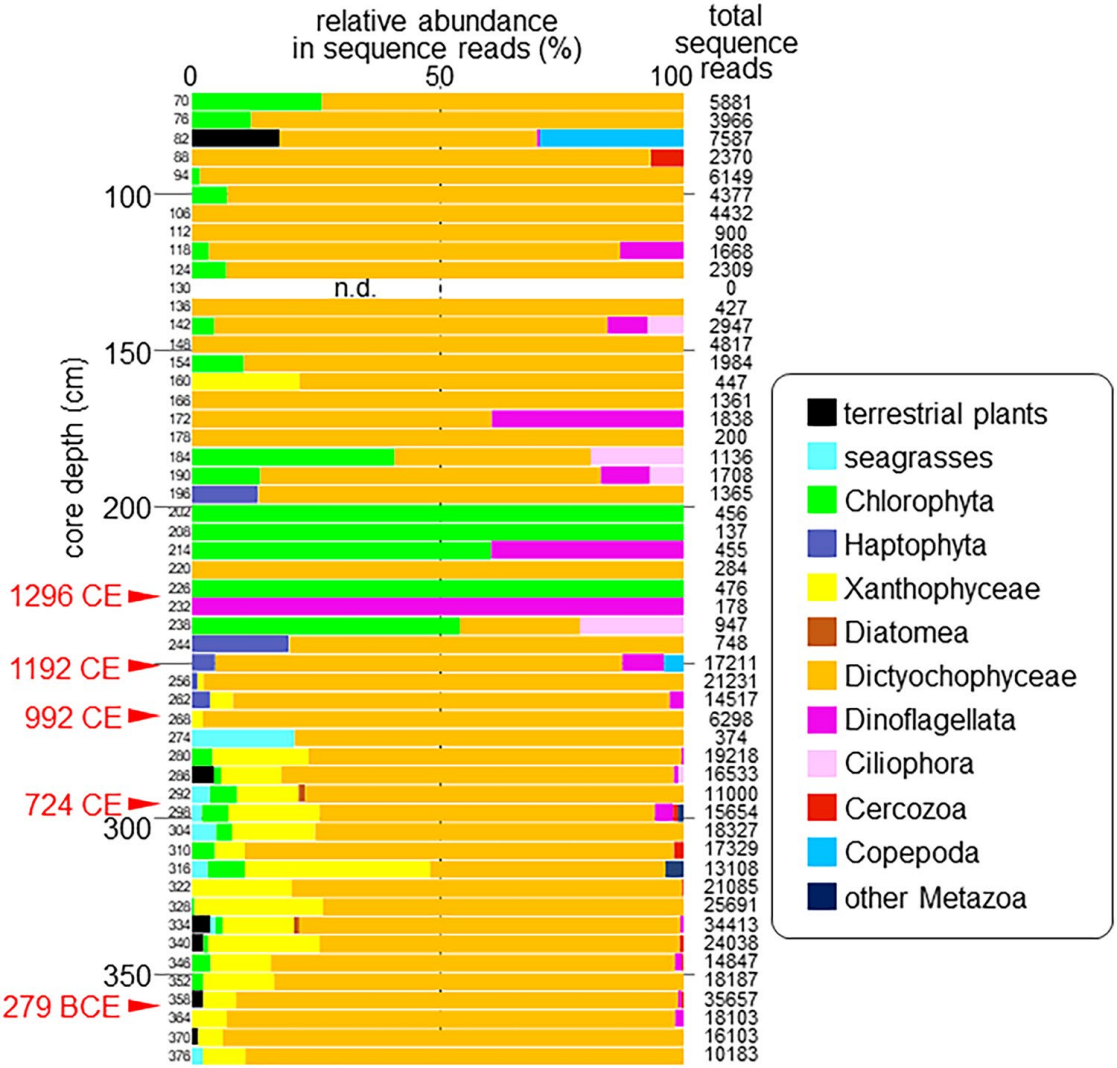
Vertical profile of higher taxa in sequence reads in the core.

In the core samples, DNA-MB detected 70 OTUs from 451,343 sequence reads; total reads ranged from 0 to 35,657 per sample (Table S3). The OTUs included about 50 genera and species belonging to 12 phyla or classes (Fig. 4, Table S3). OTUs belonging to Dictyochophyceae accounted for 81.05% of the total reads and were present in almost all core samples. Among these, the three Pedinellales clades (Fig. S2) were the most abundant. Other higher taxa included Xanthophyceae (11.54% of total reads), Chlorophyta (2.73%) and Dinoflagellata (1.20%). The taxonomic composition of the core changed dramatically (Fig. 4): Dictyochophyceae were dominant between 70 and 196 cm, although Chlorophyta and Dinoflagellata had larger proportions below 184 cm. Dictyochophyceae were dominant below 244 cm, where Xanthophyceae were also abundant.

Taxa with a limnetic–oligohaline habitat were dominant above 250 cm (Fig. 3a), although euhaline taxa were sporadically present. Below 256 cm, mesohaline–polyhaline taxa were dominant and euhaline taxa were persistently present. Taxa classified as “others” (cosmopolitan species and OTUs of undetermined habitat) were present throughout the core. The upward change in habitat composition around 250 cm corresponded to the steep decrease in sulfur dated around 1200 CE.

From these results (and cluster analyses shown in Fig. S4), we interpreted the core record as a high-salinity period followed by a transitional period and a low-salinity period (Fig. 3). It is noteworthy that the transitional period featured low total sequence reads (Fig. 4). The following taxa could be recognized as indicators of each period (Table S3): *Zostera marina* (seagrasses), *Botrydium*/*Tribonema* (Xanthophyceae) and *Pseudopedinella* sp.2 and sp.3 (Dictyochophyceae) in the high-salinity period. *Chlorokybus* (Chlorophyta) and *Woloszynskia* (Dinoflagellata) in the transitional period. *Chlamydomonas* (Chlorophyta), *Desmodesmus* (Chlorophyta), *Prorocentrum* (Dinoflagellata) and *Sinocalanus* (Copepoda) in the low-salinity period.

## Discussion

Although we believe our approach of combining ancient and modern DNA from the same site is reliable for the purpose of paleo-environmental reconstruction, it highlights some taphonomic issues related to DNA preservation. Plankton DNA was readily detected in the core, presumably due to the muddy sediment and anoxic environment of the lake floor. Preservation of DNA is closely tied to the mineral component of sediment; it is much better in clay than in quartz^19^.

Some plankton taxa such as Copepoda, Labyrinthulea and Ciliophora were common in the water samples (Fig. S1), but rare or absent in the core samples (Fig. 4), which suggests that their abundance is underestimated in the sediment analysis. The taphonomic literature has long addressed the precipitation and preservation of microfossils^20, 21^, whereas relatively little is firmly established with respect to sedaDNA^22, 23^. The decomposition of plankton DNA could occur in two different settings. Case 1: decomposition after death within the water column and during settlement. Case 2: decomposition after burial within the sediment. Considering the previous reports concerning the precipitation of microfossils^20, 21^ and the fact that the taxa detected in sediments are mostly ones that form robust cells (e.g., cysts), the majority of plankton seem to have died and decomposed within the water column and during settlement (case 1). Future studies should examine the process and context of decomposition of plankton DNA.

Reconstruction of paleo-ecosystems using DNA metabarcoding may be biased by two factors other than mineral composition that could affect the detection of DNA in sediment. (1) Some plankton taxa, such as Dinoflagellata (Alveolata) and Cercozoa (Rhizaria), contain more DNA than others^24^. (2) Some taxa that form cysts, resting spores or resting eggs may have their DNA better preserved. Dinoflagellate cysts, for example, are known to be well preserved as palynomorphs^25^. *Pseudopedinella* spp., the most detected taxa, also form robust cysts^26^. Our analyses suggest the following historical sequence of aquatic environments and ecosystems in Lake Shinji.

1. High-salinity period (256–376 cm): High-salinity water from Nakaumi Lagoon consistently filled the lake and supported marine and brackish water taxa. This period ended when the Hii River delivered large amounts of freshwater into Lake Shinji. Radiocarbon dating establishes that the end of the high-salinity period occurred shortly after 992 CE, as indicated by an abrupt change in sediment sulfur content and the habitat of plankton taxa (Fig. 3, Table S4). The Hii River is known to have shifted eastward and discharged into the lake in 1635 CE and 1639 CE^27^, but our results suggest that this event also occurred much earlier.
2. Transitional period (184–250 cm): The lag between the abrupt environmental change indicated by the sulfur record (Fig. 3b) and the completion of the change in taxonomic composition (Fig. 3a) implies that the ecosystem had an unstable status for a period of time. The age model (Fig. S3) suggests that this period lasted ca. 246 years. It may be that part of the lake retained a marine environment for a certain period, accounting for the occasional presence of marine plankton DNA in the core. Although DNA sequence reads do not directly reflect the abundance or biomass of taxa, the low total sequence reads in this period are suggestive of low plankton biomass/abundance consistent with strong salinity fluctuations that were hostile to both marine and freshwater species. There are few age calibration points in the upper part of the core, but the age model (Fig. S3) and a previous study^5^ indicate that sedimentation rates were higher after the high-salinity period, consistent with the change to a fluvial sedimentation regime.
3. Low-salinity period (70–178 cm): Salinity remained low and stable after the transitional period, and taxa adapted to low salinity constituted most of the plankton community, with occasional intrusions of marine or brackish water plankton. Although DNA preservation is affected by sediment composition, the decreased total sequence reads in this period compared to the high-salinity period is suggestive of lower production in the freshwater ecosystem than that in the marine one.

This study demonstrates that DNA-MB methods can be used to examine changes in ancient plankton communities when samples from core sediments are combined with analysis of modern water samples. Our results show that a drastic change in both ecosystem and aquatic environment, from a marine lagoon to a freshwater lake, took place ca. 1200 CE in Lake Shinji. It is noteworthy that a transitional period was clearly detected after the major change of salinity (sulfur concentration), suggesting that there was a time-lag between the environmental fluctuation and establishment of a new and stable ecosystem. Some taxa (e.g., Ciliophora, Copepoda and Labyrinthulea) have a significant presence in the modern water samples but are underrepresented in the sediment samples. This suggests that their tissues and DNA are poorly preserved in the sediment and perhaps degrade while still in the water column, leading to underestimation of their abundance from sediment analysis. Future studies should focus on this issue. Our results show that DNA-MB of the whole plankton community is an effective approach for the reconstruction of the paleo-environment.

### Supplementary Information

Supplementary Information.

## Data Availability

The datasets generated during the current study are available in the DNA Data Bank of Japan (DDBJ, INSDC member) repository (accession number DRA017262).
