# Supplementary material for: DNA metabarcoding focusing on the plankton community: an effective approach to reconstruct the paleo-environment
Source: Sci Rep. 2023 Dec 8;13:21642. doi: 10.1038/s41598-023-48367-z (PMC10703934; doi:10.1038/s41598-023-48367-z)
Supplement: Supplementary file 1 — Supplementary Information. [file 41598_2023_48367_MOESM1_ESM.pdf]

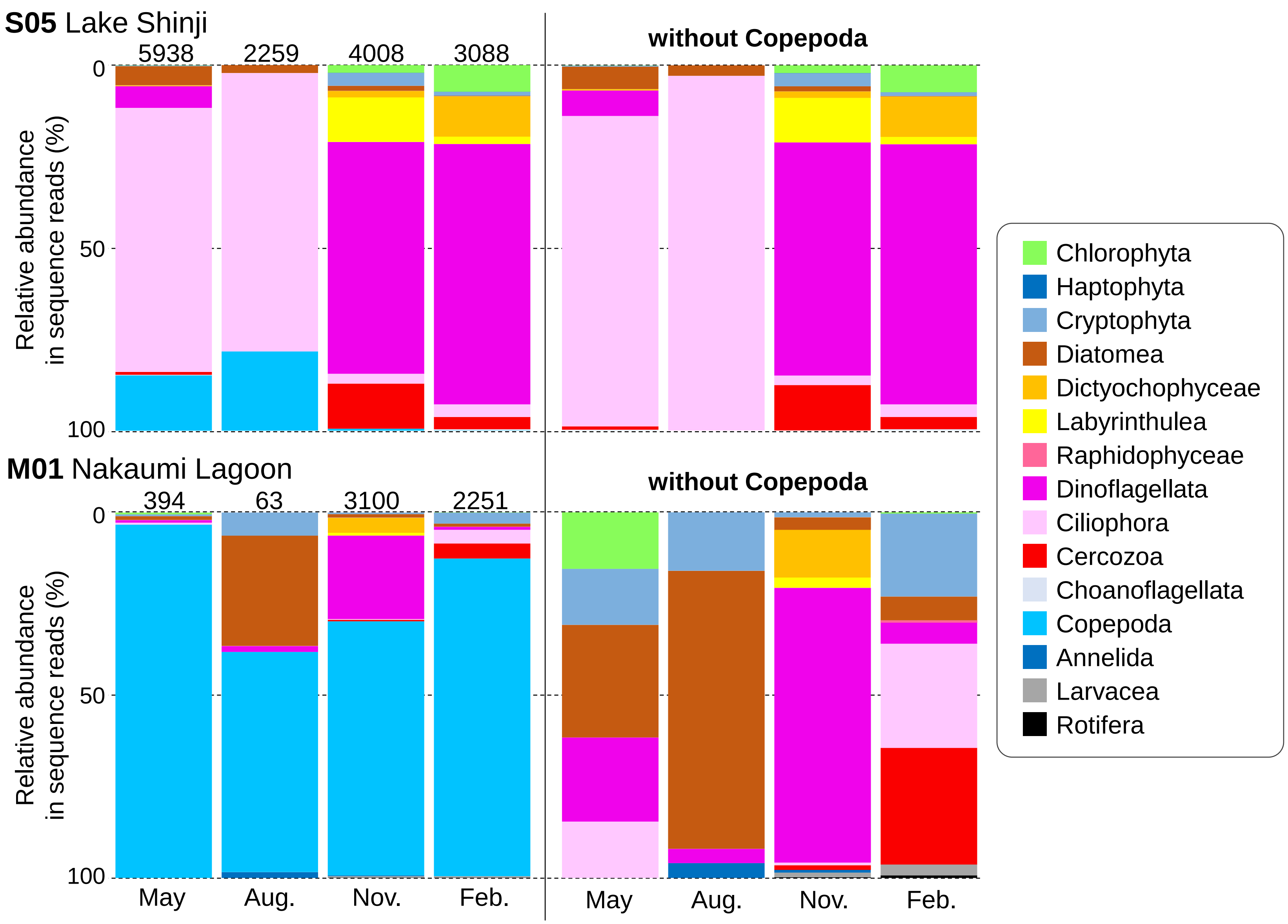

Fig. S1. Relative abundance of higher taxa in the sequence reads of the water samples. The OTUs (mainly those of plankton) are categorized according to higher taxa based on Adl et al. (2019) and Nakamura et al. (2019). Numbers above each bar graph represent the total sequence read of each sample.

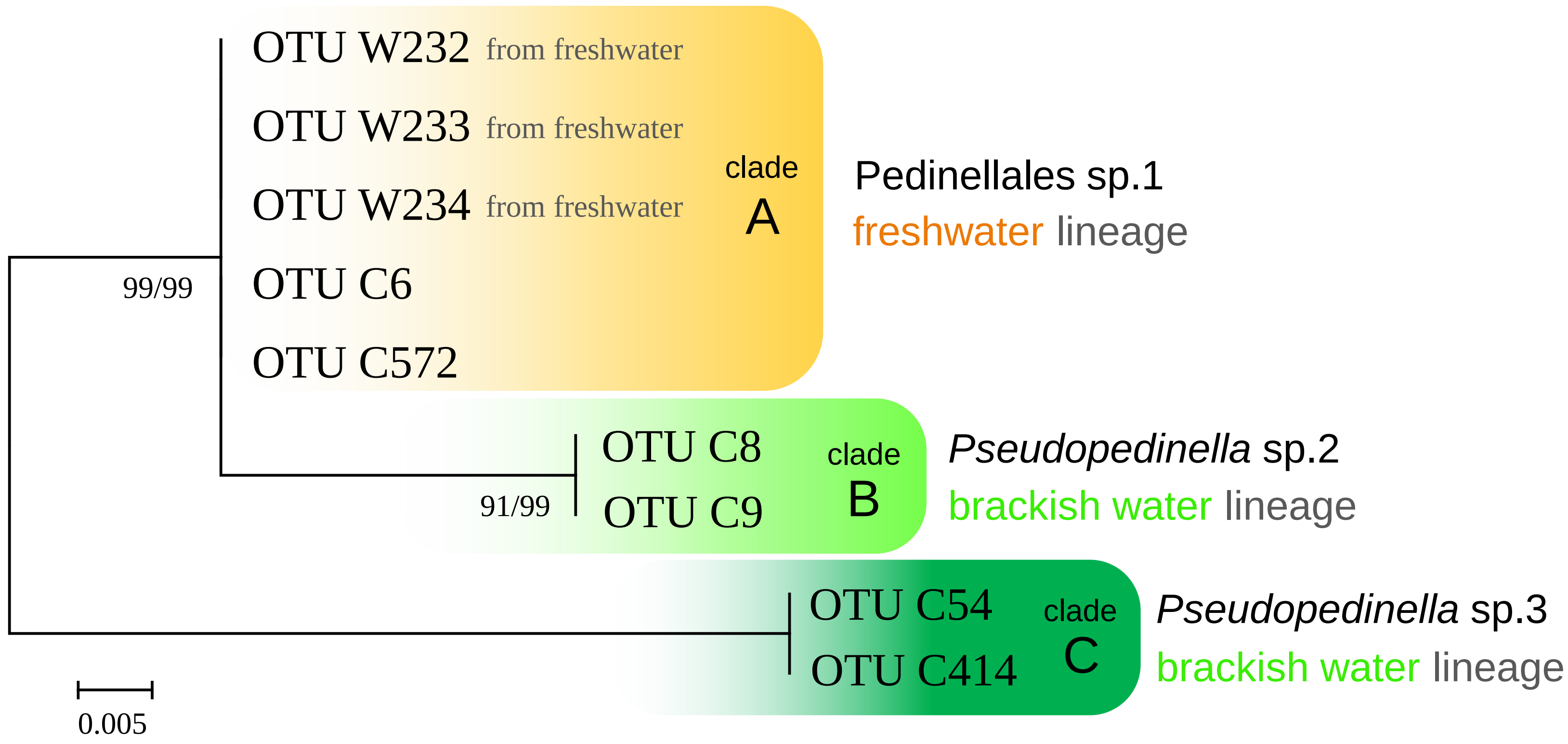

Fig. S2. Maximum-likelihood (ML) phylogenetic trees of Pedinellales (Dictyochophyceae) detected by DNA-MB of the core 21SJ (specimens with C) and of the water samples of the seasonal sampling (specimens with W). The tree was derived from partial SSU rRNA sequences. Numbers at nodes indicate 1,000 bootstrap support values (ML / Neighbor Joining). Note that the clade A is presumably a freshwater lineage judging from the included OTUs (W232, W233 and W234) which are all from freshwater environment (PSU: 0–1). The clade B and C would be brackish water lineage because the members of the genus *Pseudopedinella* (Pedinellales) are brackish water dwellers, and *Pseudopedinella* sp. has been reported in water samples from Lake Shinji (Nojiri et al. 2018).

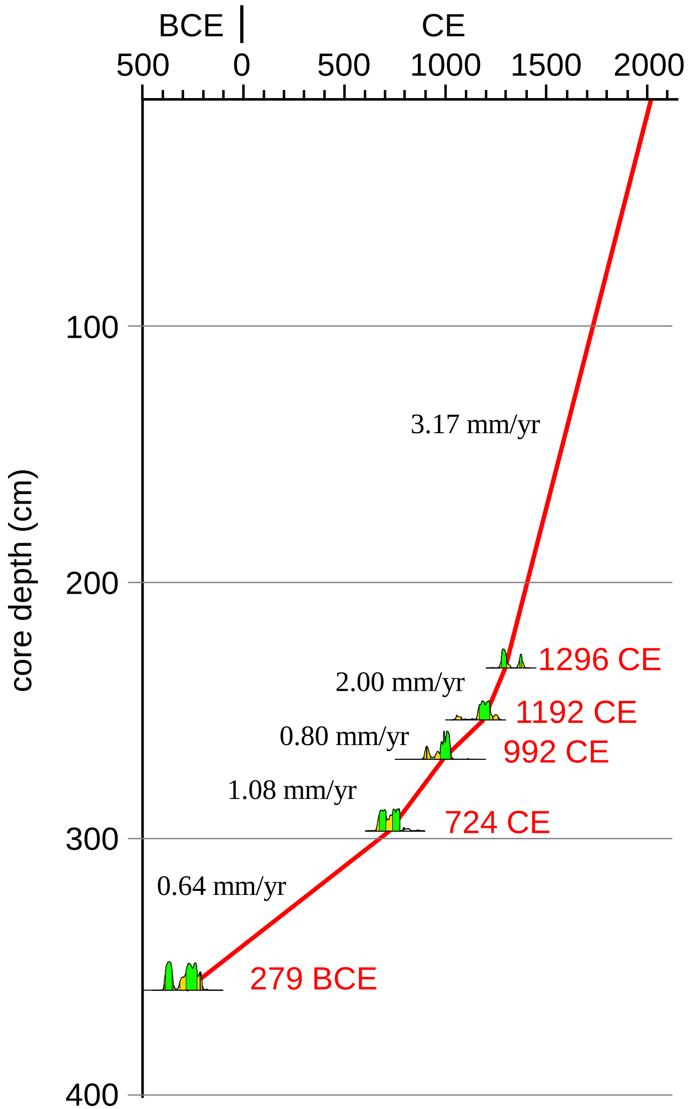

Fig S3. Age model and average sedimentation rate per year for core 21SJ.

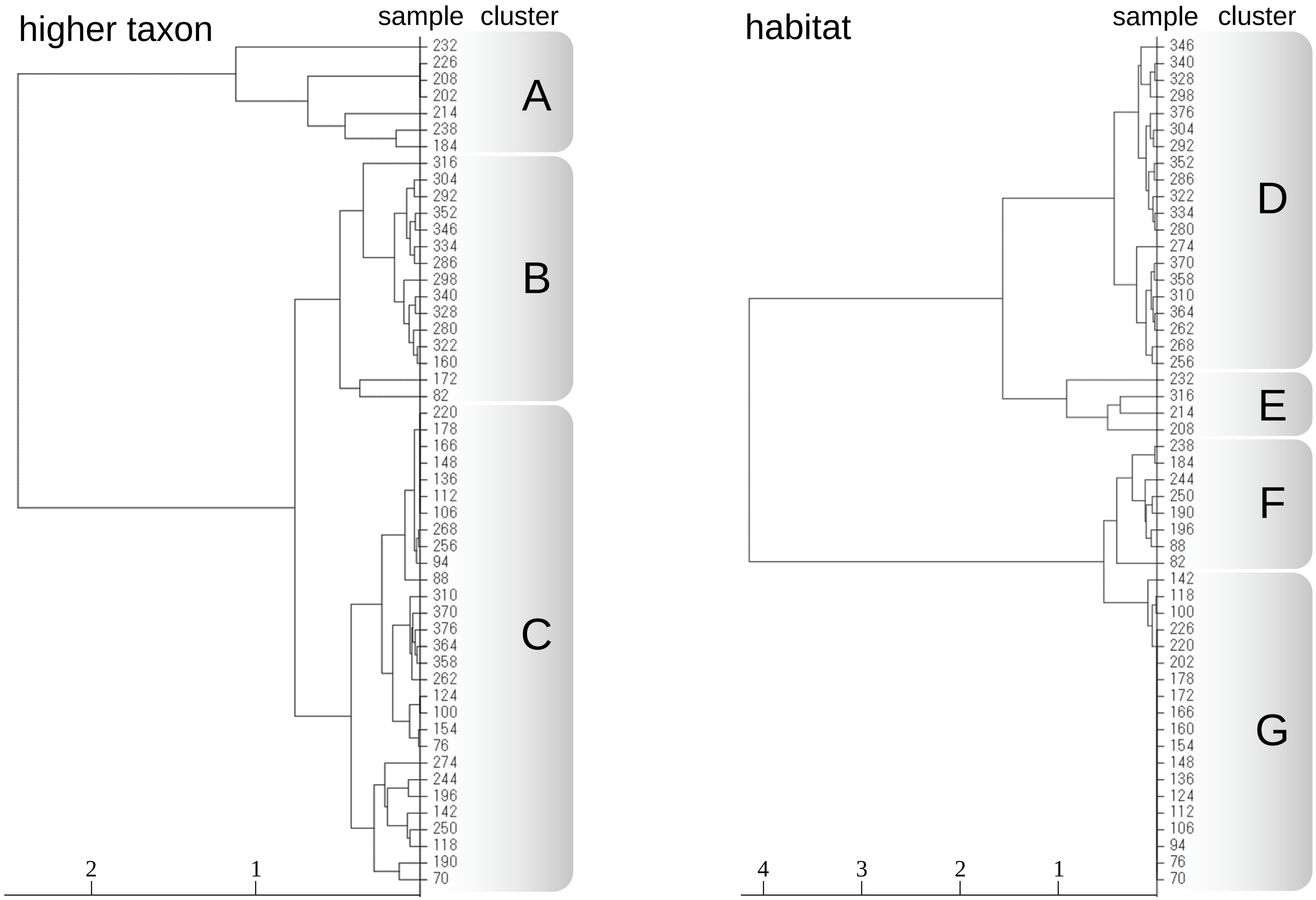

Fig. S4. Dendrograms constructed by the cluster analysis (Ward's method) based on the Euclidean distances calculated from the compositions in higher taxa and habitats of the organisms detected from the core 21SJ (Figs. 3–4, Table S3). Note that the samples names are derived from the corresponding core depths. The details about the cluster analyses are shown in the next page.

The cluster analyses were based on the composition of higher taxa and habitats. Because plankton contain taxa with different DNA copy numbers, the sequence reads of taxa detected by DNA-MB do not directly reflect the true abundance of each taxon. Considering this point, we collapsed the read numbers of OTUs into binary data (0 or 1), then calculated Euclidean distances within the resulting dataset with College Analysis ver. 6.6 software (Fukui and Hosokawa, 2004). We constructed dendrograms by Ward's method (Ward, 1963) based on the higher taxa and the habitats to visualize the differences in the core.

The results of the cluster analysis based on the composition of higher taxa revealed that samples between 184 cm and 238 cm constituted a single cluster (cluster A) distinct from two clusters in the other samples (clusters B and C) (Fig. S4). A cluster analysis based on the habitat composition showed that samples between 184 cm and 250 cm fell into two clusters (clusters E and F) distinct from two clusters in the other samples (clusters D and G).

References:

Fukui, M., & Hosokawa, M. Multi-purpose program for social system analysis 8: Canonical correlation analysis, factor analysis, utilities. *Rep. Fukuyama Heisei Univ*. 1–12 (2004). (in Japanese with English abstract)

Ward, J.H. Hierarchical groupings to optimize an objective function. *J. Amer. Stat. Assoc.* **58**, 236–244 (1963).

Table S1. Salinity classes from the Venice salinity classification (Anonymous, 1958).

salinity range (PSU)	Venice classification	common name
<0.5	limnetic	fresh water
0.5–5	oligohaline	
5–18	mesohaline	brackish water
18–30	polyhaline	
>30	euhaline	sea water

Reference

Anonymous. The Venice system for the classification of marine waters according to salinity. Limn. Ocean. 3, 346–347 (1958).

Table S2. OTUs detected by DNA-MB of the water samples taken in the seasonal investigation conducted in 2020–2021. Note that after the identification at the order-, family- and genus-level, the detected OTUs are categorized according to higher taxa based on Adl et al. (2019) and Nakamura et al. (2019).
*The habitat of each OTU was determined based on the salinity of sampling site and online taxonomic databases (e.g., Algaebase, WoRMS).

OTU	classification				habitat*	total read	May		Aug.		Nov.		Feb.	
	supergroup	phylum / class (higher taxon)	order / family	genus			M01	S05	M01	S05	M01	S05	M01	S05
**W368**	Archaeoplastida	Chlorophyta	Prasinophyceae		limnetic-oligohaline (freshwater)	142	1	2	0	0	0	1	0	138
**W369**	Archaeoplastida	Chlorophyta	Prasinophyceae		limnetic-oligohaline (freshwater)	66	1	2	0	0	0	0	0	63
**W366**	Archaeoplastida	Chlorophyta	Prasinophyceae		limnetic-oligohaline (freshwater)	14	0	0	0	0	0	0	1	13
**W370**	Archaeoplastida	Chlorophyta	Prasinophyceae		limnetic-oligohaline (freshwater)	6	0	0	0	0	0	0	0	6
**W367**	Archaeoplastida	Chlorophyta	Prasinophyceae		limnetic-oligohaline (freshwater)	5	0	0	0	0	0	0	0	5
**W372**	Archaeoplastida	Chlorophyta		*Chlamydomonas*	limnetic-oligohaline (freshwater)	57	0	0	0	0	0	57	0	0
**W373**	Archaeoplastida	Chlorophyta		*Chlamydomonas*	limnetic-oligohaline (freshwater)	25	0	0	0	0	0	25	0	0
**W359**	Haptista	Haptophyta		*Isochrysis*	limnetic-oligohaline (freshwater)	3	0	0	0	0	0	2	0	1
**W364**	Cryptista	Cryptophyta		*Geminigera*	limnetic-oligohaline (freshwater)	5	0	0	0	0	1	0	3	1
**W365**	Cryptista	Cryptophyta		*Geminigera*	limnetic-oligohaline (freshwater)	2	0	0	0	0	0	1	1	0
**W357**	Cryptista	Cryptophyta		*Katablepharis*	limnetic-oligohaline (freshwater)	111	0	0	0	0	0	87	0	24
**W358**	Cryptista	Cryptophyta		*Katablepharis*	limnetic-oligohaline (freshwater)	31	0	0	0	0	0	26	0	5
**W361**	Cryptista	Cryptophyta		*Teleaulax*	mesohaline-polyhaline	59	1	3	1	0	6	11	35	2
**W362**	Cryptista	Cryptophyta		*Teleaulax*	mesohaline-polyhaline	36	0	7	1	0	2	10	16	0
**W363**	Cryptista	Cryptophyta		*Teleaulax*	mesohaline-polyhaline	14	0	2	0	0	1	4	7	0
**W397**	Cryptista	Cryptophyta		*Teleaulax*	mesohaline-polyhaline	4	0	0	1	0	0	1	2	0
**W371**	Cryptista	Cryptophyta		other Cryptophyta	euhaline (sea water)	4	1	0	0	0	3	0	0	0
**W308**	Cryptista	Cryptophyta	Picozoa	*Picomonas*	mesohaline-polyhaline	9	0	0	1	0	0	4	3	1
**W265**	Stramenopiles	Diatomea		*Chaetoceros*	euhaline (sea water)	4	1	0	0	0	3	0	0	0
**W266**	Stramenopiles	Diatomea		*Chaetoceros*	euhaline (sea water)	2	2	0	0	0	0	0	0	0
**W258**	Stramenopiles	Diatomea		*Cyclotella*	mesohaline-polyhaline	11	0	4	0	0	0	0	6	1
**W260**	Stramenopiles	Diatomea		*Cyclotella*	mesohaline-polyhaline	9	0	2	0	0	1	0	6	0
**W259**	Stramenopiles	Diatomea		*Cyclotella*	mesohaline-polyhaline	6	0	1	0	0	0	0	5	0
**W262**	Stramenopiles	Diatomea		*Cyclotella*	mesohaline-polyhaline	151	0	122	0	5	5	18	0	1
**W264**	Stramenopiles	Diatomea		*Cyclotella*	mesohaline-polyhaline	109	0	78	1	2	4	24	0	0
**W263**	Stramenopiles	Diatomea		*Cyclotella*	mesohaline-polyhaline	76	0	56	0	4	4	11	0	1
**W249**	Stramenopiles	Diatomea		*Skeletonema*	mesohaline-polyhaline	15	1	0	0	0	14	0	0	0
**W254**	Stramenopiles	Diatomea		*Skeletonema*	mesohaline-polyhaline	65	0	35	0	30	0	0	0	0
**W250**	Stramenopiles	Diatomea		*Skeletonema*	mesohaline-polyhaline	3	0	0	0	0	1	0	2	0
**W248**	Stramenopiles	Diatomea		*Skeletonema*	mesohaline-polyhaline	4	0	0	4	0	0	0	0	0
**W255**	Stramenopiles	Diatomea		*Skeletonema*	mesohaline-polyhaline	23	0	13	0	10	0	0	0	0
**W251**	Stramenopiles	Diatomea		*Thalassiosira*	euhaline (sea water)	12	0	0	12	0	0	0	0	0
**W67**	Stramenopiles	Diatomea		other diatom	others	3	0	0	2	0	0	1	0	0
**W239**	Stramenopiles	Dictyochophyceae		*Dictyocha*	euhaline (sea water)	66	0	0	0	0	66	0	0	0
**W240**	Stramenopiles	Dictyochophyceae		*Dictyocha*	euhaline (sea water)	36	0	0	0	0	36	0	0	0
**W241**	Stramenopiles	Dictyochophyceae		*Dictyocha*	euhaline (sea water)	14	0	0	0	0	14	0	0	0
**W242**	Stramenopiles	Dictyochophyceae		*Dictyocha*	euhaline (sea water)	7	0	0	0	0	7	0	0	0
**W232**	Stramenopiles	Dictyochophyceae	Pedinellales	Pedinellales sp.1	limnetic-oligohaline (freshwater)	236	0	8	0	0	0	44	0	184
**W233**	Stramenopiles	Dictyochophyceae	Pedinellales	Pedinellales sp.1	limnetic-oligohaline (freshwater)	56	0	1	0	0	0	8	0	47
**W234**	Stramenopiles	Dictyochophyceae	Pedinellales	Pedinellales sp.1	limnetic-oligohaline (freshwater)	133	0	7	0	0	0	16	0	110
**W244**	Stramenopiles	Labyrinthulae		*Oblongichytrium*	mesohaline-polyhaline	352	0	0	0	0	19	297	0	36
**W246**	Stramenopiles	Labyrinthulae		*Oblongichytrium*	mesohaline-polyhaline	127	0	0	0	0	5	106	0	16
**W247**	Stramenopiles	Labyrinthulae		*Oblongichytrium*	mesohaline-polyhaline	60	0	0	0	0	3	51	0	6
**W245**	Stramenopiles	Labyrinthulae		*Oblongichytrium*	mesohaline-polyhaline	40	0	0	0	0	0	35	0	5
**W243**	Stramenopiles	Labyrinthulae		*Oblongichytrium*	mesohaline-polyhaline	2	0	0	0	0	0	2	0	0
**W231**	Stramenopiles	Raphidophyceae		*Chattonella*/*Heterosigma*	euhaline (sea water)	2	0	0	0	0	0	0	2	0
**W377**	Alveolata	Dinoflagellata		*Akashiwo*	euhaline (sea water)	89	0	0	0	0	89	0	0	0
**W379**	Alveolata	Dinoflagellata		*Akashiwo*	euhaline (sea water)	28	0	0	0	0	28	0	0	0
**W378**	Alveolata	Dinoflagellata		*Akashiwo*	euhaline (sea water)	9	0	0	0	0	9	0	0	0
**W374**	Alveolata	Dinoflagellata		*Amoebophrya*	mesohaline-polyhaline	141	0	3	0	0	1	30	0	107
**W375**	Alveolata	Dinoflagellata		*Amoebophrya*	mesohaline-polyhaline	87	0	1	0	0	0	21	0	65
**W376**	Alveolata	Dinoflagellata		*Amoebophrya*	mesohaline-polyhaline	69	0	0	0	0	0	19	0	50
**W380**	Alveolata	Dinoflagellata		*Gyrodinium*	mesohaline-polyhaline	16	0	13	0	0	0	0	3	0
**W382**	Alveolata	Dinoflagellata		*Gyrodinium*	mesohaline-polyhaline	5	0	3	0	0	0	0	2	0
**W381**	Alveolata	Dinoflagellata		*Gyrodinium*	mesohaline-polyhaline	4	0	4	0	0	0	0	0	0
**W393**	Alveolata	Dinoflagellata		*Heterocapsa*	mesohaline-polyhaline	2367	0	169	0	0	0	1216	0	982
**W396**	Alveolata	Dinoflagellata		*Heterocapsa*	mesohaline-polyhaline	1596	0	100	0	0	0	820	2	674
**W394**	Alveolata	Dinoflagellata		*Heterocapsa*	mesohaline-polyhaline	429	0	28	0	0	0	232	3	166
**W395**	Alveolata	Dinoflagellata		*Heterocapsa*	mesohaline-polyhaline	384	0	30	0	0	0	200	1	153
**W388**	Alveolata	Dinoflagellata		*Heterocapsa*	mesohaline-polyhaline	7	0	0	1	0	2	3	1	0
**W390**	Alveolata	Dinoflagellata		*Karlodinium*/*Gymnodinium*	euhaline (sea water)	4	2	0	0	0	2	0	0	0
**W391**	Alveolata	Dinoflagellata		*Karlodinium*/*Gymnodinium*	euhaline (sea water)	4	1	0	0	0	3	0	0	0
**W386**	Alveolata	Dinoflagellata		*Prorocentrum*	euhaline (sea water)	113	0	0	0	0	111	0	2	0
**W383**	Alveolata	Dinoflagellata		*Prorocentrum*	euhaline (sea water)	314	0	0	0	0	314	0	0	0
**W387**	Alveolata	Dinoflagellata		*Prorocentrum*	euhaline (sea water)	52	0	0	0	0	52	0	0	0
**W392**	Alveolata	Dinoflagellata		*Prorocentrum*	euhaline (sea water)	14	0	0	0	0	11	0	3	0
**W385**	Alveolata	Dinoflagellata		*Prorocentrum*	euhaline (sea water)	56	0	0	0	0	56	0	0	0
**W384**	Alveolata	Dinoflagellata		*Prorocentrum*	euhaline (sea water)	28	0	0	0	0	28	0	0	0
**W422**	Alveolata	Dinoflagellata		*Prorocentrum*/*Blastodinium*	euhaline (sea water)	3	0	0	0	0	3	0	0	0
**W401**	Alveolata	Ciliophora		*Tintinnopsis*	euhaline (sea water)	5	0	0	0	0	0	0	5	0
**W402**	Alveolata	Ciliophora		*Tintinnopsis*	euhaline (sea water)	5	0	0	0	0	0	0	5	0
**W403**	Alveolata	Ciliophora		*Tintinnopsis*	euhaline (sea water)	2	0	0	0	0	0	0	2	0
**W407**	Alveolata	Ciliophora		tintinnids	mesohaline-polyhaline	6	0	2	0	0	0	0	4	0
**W408**	Alveolata	Ciliophora		tintinnids	mesohaline-polyhaline	3	0	2	0	0	0	0	1	0
**W322**	Alveolata	Ciliophora		*Didinium*/*Dileptus*	mesohaline-polyhaline	10	0	9	0	1	0	0	0	0
**W334**	Alveolata	Ciliophora		*Mesodinium*	mesohaline-polyhaline	541	0	0	0	539	0	2	0	0
**W346**	Alveolata	Ciliophora		*Mesodinium*	mesohaline-polyhaline	41	0	4	0	8	2	1	22	4
**W352**	Alveolata	Ciliophora		*Mesodinium*	mesohaline-polyhaline	21	1	1	0	0	2	1	13	3
**W347**	Alveolata	Ciliophora		*Mesodinium*	mesohaline-polyhaline	15	0	4	0	0	2	0	6	3
**W341**	Alveolata	Ciliophora		*Mesodinium*	mesohaline-polyhaline	286	0	0	0	285	0	1	0	0
**W335**	Alveolata	Ciliophora		*Mesodinium*	mesohaline-polyhaline	245	0	0	0	243	0	2	0	0
**W323**	Alveolata	Ciliophora		*Mesodinium*	mesohaline-polyhaline	1269	0	1223	0	20	0	26	0	0
**W342**	Alveolata	Ciliophora		*Mesodinium*	mesohaline-polyhaline	184	0	0	0	182	0	2	0	0
**W348**	Alveolata	Ciliophora		*Mesodinium*	mesohaline-polyhaline	8	1	1	0	0	0	0	3	3
**W353**	Alveolata	Ciliophora		*Mesodinium*	mesohaline-polyhaline	11	0	1	0	0	0	0	8	2
**W324**	Alveolata	Ciliophora		*Mesodinium*	mesohaline-polyhaline	861	0	831	0	7	0	23	0	0
**W349**	Alveolata	Ciliophora		*Mesodinium*	mesohaline-polyhaline	4	0	0	0	0	0	0	4	0
**W354**	Alveolata	Ciliophora		*Mesodinium*	mesohaline-polyhaline	8	0	2	0	0	1	1	3	1
**W329**	Alveolata	Ciliophora		*Mesodinium*	mesohaline-polyhaline	686	0	668	0	2	0	16	0	0
**W336**	Alveolata	Ciliophora		*Mesodinium*	mesohaline-polyhaline	102	0	0	0	101	0	1	0	0
**W343**	Alveolata	Ciliophora		*Mesodinium*	mesohaline-polyhaline	84	0	0	0	84	0	0	0	0
**W337**	Alveolata	Ciliophora		*Mesodinium*	mesohaline-polyhaline	63	0	0	0	61	0	2	0	0
**W355**	Alveolata	Ciliophora		*Mesodinium*	mesohaline-polyhaline	2	0	1	0	0	0	1	0	0

**W344**	Alveolata	Ciliophora	*Mesodinium*	mesohaline-polyhaline	59	0	0	0	59	0	0	0	0
**W350**	Alveolata	Ciliophora	*Mesodinium*	mesohaline-polyhaline	3	0	0	0	1	0	0	0	2
**W338**	Alveolata	Ciliophora	*Mesodinium*	mesohaline-polyhaline	48	0	0	0	48	0	0	0	0
**W339**	Alveolata	Ciliophora	*Mesodinium*	mesohaline-polyhaline	35	0	0	0	34	0	1	0	0
**W330**	Alveolata	Ciliophora	*Mesodinium*	mesohaline-polyhaline	272	0	270	0	1	0	1	0	0
**W325**	Alveolata	Ciliophora	*Mesodinium*	mesohaline-polyhaline	235	0	226	0	4	0	5	0	0
**W331**	Alveolata	Ciliophora	*Mesodinium*	mesohaline-polyhaline	244	0	241	0	0	0	3	0	0
**W326**	Alveolata	Ciliophora	*Mesodinium*	mesohaline-polyhaline	224	0	220	0	2	0	2	0	0
**W332**	Alveolata	Ciliophora	*Mesodinium*	mesohaline-polyhaline	179	0	176	0	0	0	3	0	0
**W327**	Alveolata	Ciliophora	*Mesodinium*	mesohaline-polyhaline	185	0	176	0	3	0	6	0	0
**W328**	Alveolata	Ciliophora	*Mesodinium*	mesohaline-polyhaline	115	0	107	0	2	0	6	0	0
**W333**	Alveolata	Ciliophora	*Mesodinium*	mesohaline-polyhaline	123	0	120	0	2	0	1	0	0
**W345**	Alveolata	Ciliophora	*Mesodinium*	mesohaline-polyhaline	11	0	0	0	11	0	0	0	0
**W340**	Alveolata	Ciliophora	*Mesodinium*	mesohaline-polyhaline	17	0	0	0	17	0	0	0	0
**W414**	Alveolata	Ciliophora	*Strombidium*	mesohaline-polyhaline	10	0	0	0	0	0	0	4	6
**W415**	Alveolata	Ciliophora	*Strombidium*	mesohaline-polyhaline	11	0	0	0	0	0	0	3	8
**W417**	Alveolata	Ciliophora	*Strombidium*	mesohaline-polyhaline	3	0	1	0	0	0	0	0	2
**W416**	Alveolata	Ciliophora	*Strombidium*	mesohaline-polyhaline	9	0	0	0	0	0	0	0	9
**W420**	Alveolata	Ciliophora	*Strombidium*	mesohaline-polyhaline	39	0	0	0	0	0	0	0	39
**W418**	Alveolata	Ciliophora	*Strombidium*	mesohaline-polyhaline	2	0	0	0	0	0	0	1	1
**W421**	Alveolata	Ciliophora	*Strombidium*	mesohaline-polyhaline	25	0	0	0	0	0	0	0	25
**W292**	Rhizaria	Cercozoa	*Cryothecomonas*	limnetic-oligohaline (freshwater)	24	0	0	0	0	0	0	3	21
**W294**	Rhizaria	Cercozoa	*Cryothecomonas*	limnetic-oligohaline (freshwater)	22	0	0	0	0	0	0	3	19
**W282**	Rhizaria	Cercozoa	*Cryothecomonas*	limnetic-oligohaline (freshwater)	25	0	0	0	0	0	0	0	25
**W293**	Rhizaria	Cercozoa	*Cryothecomonas*	limnetic-oligohaline (freshwater)	2	0	0	0	0	0	0	0	2
**W284**	Rhizaria	Cercozoa	*Cryothecomonas*	limnetic-oligohaline (freshwater)	16	0	0	0	0	0	0	0	16
**W283**	Rhizaria	Cercozoa	*Cryothecomonas*	limnetic-oligohaline (freshwater)	12	0	0	0	0	0	0	0	12
**W288**	Rhizaria	Cercozoa	*Cryothecomonas*	limnetic-oligohaline (freshwater)	4	0	0	0	0	0	0	0	4
**W281**	Rhizaria	Cercozoa	*Euglypha*	euhaline (sea water)	10	0	0	0	0	10	0	0	0
**W289**	Rhizaria	Cercozoa	*Rhogostoma*	limnetic-oligohaline (freshwater)	340	0	32	0	0	2	306	0	0
**W295**	Rhizaria	Cercozoa	other Cercozoa	others	79	0	0	0	0	0	19	57	3
**W291**	Rhizaria	Cercozoa	other Cercozoa	others	138	0	11	0	0	0	127	0	0
**W296**	Rhizaria	Cercozoa	other Cercozoa	others	35	0	0	0	0	0	10	25	0
**W290**	Rhizaria	Cercozoa	other Cercozoa	others	32	0	2	0	0	0	30	0	0
**W297**	Rhizaria	Cercozoa	other Cercozoa	others	9	0	0	0	0	0	2	6	1
**W306**	Opisthokonta	Choanoflagellata		limnetic-oligohaline (freshwater)	23	0	12	0	0	0	0	0	11
**W315**	Opisthokonta	Copepoda	Calanoida	limnetic-oligohaline (freshwater)	611	0	357	0	243	0	9	0	2
**W317**	Opisthokonta	Copepoda	Calanoida	limnetic-oligohaline (freshwater)	361	0	258	0	94	0	9	0	0
**W316**	Opisthokonta	Copepoda	Calanoida	limnetic-oligohaline (freshwater)	351	1	235	0	111	0	3	0	1
**W311**	Opisthokonta	Copepoda	Calanoida	euhaline (sea water)	144	0	0	0	0	6	0	138	0
**W312**	Opisthokonta	Copepoda	Calanoida	euhaline (sea water)	86	0	0	0	0	3	0	83	0
**W318**	Opisthokonta	Copepoda	Calanoida	limnetic-oligohaline (freshwater)	34	0	22	0	10	0	2	0	0
**W313**	Opisthokonta	Copepoda	Calanoida	euhaline (sea water)	12	0	0	0	0	0	0	12	0
**W314**	Opisthokonta	Copepoda	Calanoida	euhaline (sea water)	2	0	0	2	0	0	0	0	0
**W275**	Opisthokonta	Copepoda	Oithonida	mesohaline-polyhaline	1520	228	14	19	0	978	0	281	0
**W276**	Opisthokonta	Copepoda	Oithonida	mesohaline-polyhaline	787	80	7	9	0	544	1	146	0
**W278**	Opisthokonta	Copepoda	Oithonida	mesohaline-polyhaline	589	52	7	7	0	404	0	119	0
**W279**	Opisthokonta	Copepoda	Oithonida	mesohaline-polyhaline	106	12	1	1	0	75	0	17	0
**W277**	Opisthokonta	Copepoda	Oithonida	mesohaline-polyhaline	52	8	0	0	0	30	0	14	0
**W280**	Opisthokonta	Copepoda	Oithonida	mesohaline-polyhaline	2	0	0	0	2	0	0	0	0
**W267**	Opisthokonta	Copepoda	*Acartia*	euhaline (sea water)	24	0	0	0	0	0	0	24	0
**W269**	Opisthokonta	Copepoda	*Acartia*	euhaline (sea water)	8	0	0	0	0	0	0	8	0
**W268**	Opisthokonta	Copepoda	*Acartia*	euhaline (sea water)	6	0	0	0	0	0	0	6	0
**W271**	Opisthokonta	Copepoda	*Paracalanus*	euhaline (sea water)	823	0	0	0	0	73	0	750	0
**W273**	Opisthokonta	Copepoda	*Paracalanus*	euhaline (sea water)	360	0	0	0	0	35	0	325	0
**W272**	Opisthokonta	Copepoda	*Paracalanus*	euhaline (sea water)	41	0	0	0	0	8	0	33	0
**W309**	Opisthokonta	Copepoda	*Pseudodiaptomus*	limnetic-oligohaline (freshwater)	27	0	0	0	27	0	0	0	0
**W310**	Opisthokonta	Copepoda	*Pseudodiaptomus*	limnetic-oligohaline (freshwater)	4	0	0	0	4	0	0	0	0
**W319**	Opisthokonta	Annelida	*Prionospio*	euhaline (sea water)	8	0	0	1	0	7	0	0	0
**W298**	Opisthokonta	Larvacea	*Oikopleura*	euhaline (sea water)	21	0	0	0	0	12	0	9	0
**W303**	Opisthokonta	Rotifera	*Synchaeta*	euhaline (sea water)	4	0	0	0	0	2	0	2	0
					total read	394	5938	63	2259	3100	4008	2251	3088

Table S3. OTUs detected by DNA-MB of the sedimentary core 21SJ. Note that after the identification at the order-, family- and genus-level, the detected OTUs are categorized according to higher taxa based on Adl et al. (2019) and Nakamura et al. (2019).
*: The habitat of each OTU was determined based on the online taxonomic databases (e.g., Algaebase, WoRMS) and the results from water samples taken in the seasonal investigation (Table S2).
**: OTUs belonging to Pedinellales (Dictyochophyceae). The habitats of these OTUs were determined by constructing a phylogenetic tree (Fig. S2) including other OTUs detected from the water samples (Fig. S1, Table S2).

OTU	classification			habitat*	total read	core depth (upper depth, cm)																		
	phylum/class (higher taxon)	order/family	genus/species			70	76	82	88	94	100	106	112	118	124	130	136	142	148	154	160	166	172	178
C768	terrestrial plants	Poaceae		others	726	0	0	0	0	0	0	0	0	0	0	0	0	0	0	0	0	0	0	0
C101	terrestrial plants	Poaceae		others	3185	0	0	1344	0	0	0	0	0	0	0	0	0	0	0	0	0	0	0	0
C735	terrestrial plants		other terrestrial plants	others	812	0	0	0	0	0	0	0	0	0	0	0	0	0	0	0	0	0	0	0
C1118	terrestrial plants	Cannabaceae		others	205	0	0	0	0	0	0	0	0	0	0	0	0	0	0	0	0	0	0	0
C481	seagrasses		*Zostera marina*	euhaline (sea water)	1733	0	0	0	0	0	0	0	0	0	0	0	0	0	0	0	0	0	0	0
C686	seagrasses		*Zostera marina*	euhaline (sea water)	773	0	0	0	0	0	0	0	0	0	0	0	0	0	0	0	0	0	0	0
C1089	seagrasses		*Zostera marina*	euhaline (sea water)	227	0	0	0	0	0	0	0	0	0	0	0	0	0	0	0	0	0	0	0
C490	Chlorophyta		*Chlorokybus*	limnetic-oligohaline (freshwater)	2117	0	0	0	0	0	0	0	0	0	160	0	0	0	0	0	0	0	0	0
C1224	Chlorophyta	Prasinophyceae	*Nephroselmis*	euhaline (sea water)	123	0	0	0	0	0	0	0	0	0	0	0	0	0	0	0	0	0	0	0
C890	Chlorophyta	Prasinophyceae	*Pyramimonas*	others	483	0	0	0	0	0	95	0	0	57	0	0	0	0	0	0	0	0	0	0
C1052	Chlorophyta	Prasinophyceae	*Pyramimonas*	others	277	0	0	0	0	0	0	0	0	0	0	0	0	0	0	0	0	0	0	0
C854	Chlorophyta	Prasinophyceae		others	555	0	0	0	0	0	0	0	0	0	0	0	0	0	0	0	0	0	0	0
C946	Chlorophyta		*Bathycoccus*	euhaline (sea water)	389	0	0	0	0	0	0	0	0	0	0	0	0	0	0	0	0	0	0	0
C344	Chlorophyta		*Chlamydomonas*	limnetic-oligohaline (freshwater)	2242	1554	478	0	0	0	0	0	0	0	0	0	0	0	0	210	0	0	0	0
C1100	Chlorophyta		*Chloricystis*	others	218	0	0	0	0	0	0	0	0	0	0	0	0	0	0	0	0	0	0	0
C951	Chlorophyta		*Choricystis*	others	383	0	0	0	0	0	0	0	0	0	0	0	0	0	0	0	0	0	0	0
C172	Chlorophyta		*Desmodesmus*	limnetic-oligohaline (freshwater)	318	0	0	0	0	98	220	0	0	0	0	0	0	0	0	0	0	0	0	0
C1127	Chlorophyta		*Lagerheimia*	limnetic-oligohaline (freshwater)	196	0	0	0	0	0	0	0	0	0	0	0	0	0	0	0	0	0	0	0
C1110	Chlorophyta		*Mantoniella*	euhaline (sea water)	208	0	0	0	0	0	0	0	0	0	0	0	0	0	0	0	0	0	0	0
C933	Chlorophyta		*Nannochloris*	euhaline (sea water)	410	0	0	0	0	0	0	0	0	0	0	0	0	0	0	0	0	0	0	0
C434	Chlorophyta		*Nannochlorum*	euhaline (sea water)	2580	0	0	0	0	0	0	0	0	0	0	0	0	0	0	0	0	0	0	0
C833	Chlorophyta		*Pseudochloris*	euhaline (sea water)	589	0	0	0	0	0	0	0	0	0	0	0	0	0	0	0	0	0	0	0
C1108	Chlorophyta		*Pseudochloris*	euhaline (sea water)	210	0	0	0	0	0	0	0	0	0	0	0	0	0	0	0	0	0	0	0
C1206	Chlorophyta		*Trochiscia*	limnetic-oligohaline (freshwater)	133	0	0	0	0	0	0	0	0	0	0	0	0	133	0	0	0	0	0	0
C928	Chlorophyta		*Nanochlorum/Picochlorum*	euhaline (sea water)	414	0	0	0	0	0	0	0	0	0	0	0	0	0	0	0	0	0	0	0
C972	Chlorophyta		*Nanochlorum/Picochlorum*	euhaline (sea water)	360	0	0	0	0	0	0	0	0	0	0	0	0	0	0	0	0	0	0	0
C1232	Chlorophyta		*Oocystis*	others	117	0	0	0	0	0	0	0	0	0	0	0	0	0	0	0	0	0	0	0
C511	Haptophyta		*Pavlova*	euhaline (sea water)	1931	0	0	0	0	0	0	0	0	0	0	0	0	0	0	0	0	0	0	0
C33	Xanthophyceae		*Botrydium/Tribonema*	others	51295	0	0	0	0	0	0	0	0	0	0	0	0	0	0	0	0	0	0	0
C800	Xanthophyceae		*Botrydium/Tribonema*	others	674	0	0	0	0	0	0	0	0	0	0	0	0	0	0	0	0	0	0	0
C1255	Xanthophyceae		*Monodus subterranea*	limnetic-oligohaline (freshwater)	98	0	0	0	0	0	0	0	0	0	0	0	0	0	0	0	98	0	0	0
C953	Diatomea	centric	*Chaetoceros*	euhaline (sea water)	381	0	0	0	0	0	0	0	0	0	0	0	0	0	0	0	0	0	0	0
C1195	Diatomea	pennate	*Pleurosigma*	euhaline (sea water)	142	0	0	0	0	0	0	0	0	0	0	0	0	0	0	0	0	0	0	0
**C6****	**Dictyochophyceae**	**Pedinellales**	**Pedinellales sp.1**	**limnetic-oligohaline (freshwater)**	**65727**	**4327**	**3488**	**3981**	**2210**	**6051**	**4062**	**4432**	**900**	**1396**	**2149**	**0**	**427**	**2360**	**4817**	**1774**	**0**	**1361**	**1121**	**200**
**C572****	**Dictyochophyceae**	**Pedinellales**	**Pedinellales sp.1**	**limnetic-oligohaline (freshwater)**	**633**	**0**	**0**	**0**	**0**	**0**	**0**	**0**	**0**	**0**	**0**	**0**	**0**	**0**	**0**	**0**	**349**	**0**	**0**	**0**
**C8****	**Dictyochophyceae**	**Pedinellales**	***Pseudopedinella* sp.2**	**mesohaline-polyhaline**	**133884**	**0**	**0**	**0**	**0**	**0**	**0**	**0**	**0**	**0**	**0**	**0**	**0**	**0**	**0**	**0**	**0**	**0**	**0**	**0**
**C9****	**Dictyochophyceae**	**Pedinellales**	***Pseudopedinella* sp.2**	**mesohaline-polyhaline**	**126653**	**0**	**0**	**0**	**0**	**0**	**0**	**0**	**0**	**0**	**0**	**0**	**0**	**0**	**0**	**0**	**0**	**0**	**0**	**0**
**C54****	**Dictyochophyceae**	**Pedinellales**	***Pseudopedinella* sp.3**	**mesohaline-polyhaline**	**29546**	**0**	**0**	**0**	**0**	**0**	**0**	**0**	**0**	**0**	**0**	**0**	**0**	**0**	**0**	**0**	**0**	**0**	**0**	**0**
**C414****	**Dictyochophyceae**	**Pedinellales**	***Pseudopedinella* sp.3**	**mesohaline-polyhaline**	**2745**	**0**	**0**	**0**	**0**	**0**	**0**	**0**	**0**	**0**	**0**	**0**	**0**	**0**	**0**	**0**	**0**	**0**	**0**	**0**
C363	Dictyochophyceae		*Pteridomonas danica*	euhaline (sea water)	3283	0	0	0	0	0	0	0	0	0	0	0	0	0	0	0	0	0	0	0
C528	Dictyochophyceae		*Pteridomonas danica*	euhaline (sea water)	1837	0	0	0	0	0	0	0	0	0	0	0	0	0	0	0	0	0	0	0
C736	Dictyochophyceae		*Pteridomonas danica*	euhaline (sea water)	811	0	0	0	0	0	0	0	0	0	0	0	0	0	0	0	0	0	0	0
C792	Dictyochophyceae		*Pteridomonas danica*	euhaline (sea water)	682	0	0	0	0	0	0	0	0	0	0	0	0	0	0	0	0	0	0	0
C1047	Dinoflagellata		*Amoebophrya*	euhaline (sea water)	279	0	0	0	0	0	0	0	0	0	0	0	0	0	0	0	0	0	0	0
C1070	Dinoflagellata		*Amoebophrya*	euhaline (sea water)	254	0	0	0	0	0	0	0	0	0	0	0	0	0	0	0	0	0	0	0
C936	Dinoflagellata		*Blastodinium*	euhaline (sea water)	406	0	0	0	0	0	0	0	0	0	0	0	0	0	0	0	0	0	0	0
C1091	Dinoflagellata		*Blastodinium*	euhaline (sea water)	226	0	0	0	0	0	0	0	0	0	0	0	0	0	0	0	0	0	0	0
C1032	Dinoflagellata		*Gonyaulax*	euhaline (sea water)	294	0	0	0	0	0	0	0	0	0	0	0	0	0	0	0	0	0	0	0
C628	Dinoflagellata	Peridiniales		limnetic-oligohaline (freshwater)	1222	0	0	48	0	0	0	0	0	215	0	0	0	242	0	0	0	0	717	0
C1106	Dinoflagellata		*Pfiesteria*	euhaline (sea water)	214	0	0	0	0	0	0	0	0	0	0	0	0	0	0	0	0	0	0	0
C1156	Dinoflagellata		*Pfiesteria*	euhaline (sea water)	172	0	0	0	0	0	0	0	0	0	0	0	0	0	0	0	0	0	0	0
C1151	Dinoflagellata		*Pheopolykrikos*	euhaline (sea water)	178	0	0	0	0	0	0	0	0	0	0	0	0	0	0	0	0	0	0	0
C1021	Dinoflagellata		*Prorocentrum*	euhaline (sea water)	307	0	0	0	0	0	0	0	0	0	0	0	0	0	0	0	0	0	0	0
C885	Dinoflagellata		*Prorocentrum*	euhaline (sea water)	215	0	0	0	0	0	0	0	0	0	0	0	0	0	0	0	0	0	0	0
C1150	Dinoflagellata		*Protoceratium*	euhaline (sea water)	178	0	0	0	0	0	0	0	0	0	0	0	0	0	0	0	0	0	0	0
C580	Dinoflagellata		*Woloszynskia*	others	1462	0	0	0	0	0	0	0	0	0	0	0	0	0	0	0	0	0	0	0
C918	Ciliophora	Ciliata		others	427	0	0	0	0	0	0	0	0	0	0	0	0	212	0	0	0	0	0	0
C1123	Ciliophora	Ciliata		others	199	0	0	0	0	0	0	0	0	0	0	0	0	0	0	0	0	0	0	0
C1235	Ciliophora	Ciliata		others	116	0	0	0	0	0	0	0	0	0	0	0	0	0	0	0	0	0	0	0
C1142	Ciliophora		*Plagiocampa*	euhaline (sea water)	184	0	0	0	0	0	0	0	0	0	0	0	0	0	0	0	0	0	0	0
C1293	Cercozoa	Chlorarachniophyta	*Lotharella*	euhaline (sea water)	56	0	0	0	0	0	0	0	0	0	0	0	0	0	0	0	0	0	0	0
C982	Cercozoa		*Cercomonas*	others	350	0	0	0	0	0	0	0	0	0	0	0	0	0	0	0	0	0	0	0
C1175	Cercozoa		*Paracercomonas*	euhaline (sea water)	157	0	0	0	0	0	0	0	0	0	0	0	0	0	0	0	0	0	0	0
C1085	Cercozoa	Phaeodaria		euhaline (sea water)	160	0	0	0	160	0	0	0	0	0	0	0	0	0	0	0	0	0	0	0
C1117	Cercozoa		other Cercozoa	euhaline (sea water)	206	0	0	0	0	0	0	0	0	0	0	0	0	0	0	0	0	0	0	0
C1125	Cercozoa		other Cercozoa	euhaline (sea water)	198	0	0	0	0	0	0	0	0	0	0	0	0	0	0	0	0	0	0	0
C879	other Metazoa	Platyhelminthes	*Plerurus*	euhaline (sea water)	496	0	0	0	0	0	0	0	0	0	0	0	0	0	0	0	0	0	0	0
C1132	other Metazoa	Ctenophora	*Dryodora*	euhaline (sea water)	190	0	0	0	0	0	0	0	0	0	0	0	0	0	0	0	0	0	0	0
C798	other Metazoa	Copepoda	*oithona*	mesohaline-polyhaline	678	0	0	0	0	0	0	0	0	0	0	0	0	0	0	0	0	0	0	0
C98	other Metazoa	Copepoda	*Sinocalanus*	mesohaline-polyhaline	2214	0	0	2214	0	0	0	0	0	0	0	0	0	0	0	0	0	0	0	0
					total read	5881	3966	7587	2370	6149	4377	4432	900	1668	2309	0	427	2947	4817	1984	447	1361	1838	200

184	190	196	202	208	214	220	226	232	238	244	250	256	262	268	274	280	286	292	298	304	310	316	322	328	334	340	346	352	358	364	370	376
0	0	0	0	0	0	0	0	0	0	0	0	0	0	0	0	0	726	0	0	0	0	0	0	0	0	0	0	0	0	0	0	0
0	0	0	0	0	0	0	0	0	0	0	0	0	0	0	0	0	0	0	0	0	0	0	0	0	1284	557	0	0	0	0	0	0
0	0	0	0	0	0	0	0	0	0	0	0	0	0	0	0	0	0	0	0	0	0	0	0	0	0	0	0	0	812	0	0	0
0	0	0	0	0	0	0	0	0	0	0	0	0	0	0	0	0	0	0	0	0	0	0	0	0	0	0	0	0	0	0	205	0
0	0	0	0	0	0	0	0	0	0	0	0	0	0	0	78	0	0	0	318	907	0	430	0	0	0	0	0	0	0	0	0	0
0	0	0	0	0	0	0	0	0	0	0	0	0	0	0	0	0	0	407	0	0	0	0	0	0	366	0	0	0	0	0	0	0
0	0	0	0	0	0	0	0	0	0	0	0	0	0	0	0	0	0	0	0	0	0	0	0	0	0	0	0	0	0	0	0	227
468	237	0	260	0	0	0	476	0	516	0	0	0	0	0	0	0	0	0	0	0	0	0	0	0	0	0	0	0	0	0	0	0
0	0	0	0	0	0	0	0	0	0	0	0	0	0	0	0	0	0	0	0	0	0	0	0	123	0	0	0	0	0	0	0	0
0	0	0	0	137	0	0	0	0	0	0	0	0	0	0	0	0	42	0	0	0	0	152	0	0	0	0	0	0	0	0	0	0
0	0	0	0	0	277	0	0	0	0	0	0	0	0	0	0	0	0	0	0	0	0	0	0	0	0	0	0	0	0	0	0	0
0	0	0	0	0	0	0	0	0	0	0	0	0	0	0	0	0	0	0	167	0	388	0	0	0	0	0	0	0	0	0	0	0
0	0	0	0	0	0	0	0	0	0	0	0	0	0	0	0	389	0	0	0	0	0	0	0	0	0	0	0	0	0	0	0	0
0	0	0	0	0	0	0	0	0	0	0	0	0	0	0	0	0	0	0	0	0	0	0	0	0	0	0	0	0	0	0	0	0
0	0	0	0	0	0	0	0	0	0	0	0	0	0	0	0	0	0	0	0	0	0	0	0	0	0	218	0	0	0	0	0	0
0	0	0	0	0	0	0	0	0	0	0	0	0	0	0	0	0	0	0	0	0	0	0	0	0	383	0	0	0	0	0	0	0
0	0	0	0	0	0	0	0	0	0	0	0	0	0	0	0	0	0	0	0	0	0	0	0	0	0	0	0	0	0	0	0	0
0	0	0	196	0	0	0	0	0	0	0	0	0	0	0	0	0	0	0	0	0	0	0	0	0	0	0	0	0	0	0	0	0
0	0	0	0	0	0	0	0	0	0	0	0	0	0	0	0	0	0	0	0	0	0	0	0	0	0	0	208	0	0	0	0	0
0	0	0	0	0	0	0	0	0	0	0	0	0	0	0	0	0	0	0	0	0	0	0	0	0	0	0	0	410	0	0	0	0
0	0	0	0	0	0	0	0	0	0	0	0	0	0	0	0	410	0	601	690	0	0	879	0	0	0	0	0	0	0	0	0	0
0	0	0	0	0	0	0	0	0	0	0	0	0	0	0	0	0	0	0	0	589	0	0	0	0	0	0	0	0	0	0	0	0
0	0	0	0	0	0	0	0	0	0	0	0	0	0	0	0	0	210	0	0	0	0	0	0	0	0	0	0	0	0	0	0	0
0	0	0	0	0	0	0	0	0	0	0	0	0	0	0	0	0	0	0	0	0	0	0	0	0	0	0	0	0	0	0	0	0
0	0	0	0	0	0	0	0	0	0	0	0	0	0	0	0	0	0	0	0	0	414	0	0	0	0	0	0	0	0	0	0	0
0	0	0	0	0	0	0	0	0	0	0	0	0	0	0	0	0	0	0	0	0	0	0	0	0	0	0	360	0	0	0	0	0
0	0	0	0	0	0	0	0	0	0	0	0	0	0	0	0	0	0	0	0	0	0	0	0	0	117	0	0	0	0	0	0	0
0	0	184	0	0	0	0	0	0	0	148	816	246	537	0	0	0	0	0	0	0	0	0	0	0	0	0	0	0	0	0	0	0
0	0	0	0	0	0	0	0	0	0	0	0	252	0	140	0	3750	2028	1381	2929	3092	1063	5144	4297	6739	5007	5503	1837	2652	2442	1309	835	895
0	0	0	0	0	0	0	0	0	0	0	0	0	674	0	0	0	0	0	0	0	0	0	0	0	0	0	0	0	0	0	0	0
0	0	0	0	0	0	0	0	0	0	0	0	0	0	0	0	0	0	0	0	0	0	0	0	0	0	0	0	0	0	0	0	0
0	0	0	0	0	0	0	0	0	0	0	0	0	0	0	0	0	0	0	0	0	0	0	0	0	381	0	0	0	0	0	0	0
0	0	0	0	0	0	0	0	0	0	0	0	0	0	0	0	0	0	142	0	0	0	0	0	0	0	0	0	0	0	0	0	0
453	1183	1181	0	0	0	0	0	0	232	600	14255	0	0	0	0	0	0	0	0	0	0	0	743	0	0	0	2024	0	0	0	0	0
0	0	0	0	0	0	284	0	0	0	0	0	0	0	0	0	0	0	0	0	0	0	0	0	0	0	0	0	0	0	0	0	0
0	0	0	0	0	0	0	0	0	0	0	0	0	9543	6158	296	14228	12664	8469	10815	13739	11737	5813	2232	0	0	0	0	0	17214	9734	6491	4751
0	0	0	0	0	0	0	0	0	0	0	0	13348	3357	0	0	0	0	0	0	0	2865	0	13210	12896	18276	11935	6089	13578	13081	6071	8373	3574
0	0	0	0	0	0	0	0	0	0	0	0	6574	0	0	0	0	0	0	0	0	0	0	14	4370	8373	4597	4071	1547	0	0	0	0
0	0	0	0	0	0	0	0	0	0	0	0	0	0	0	0	249	539	0	0	0	260	0	0	0	0	0	0	0	1697	0	0	0
0	0	0	0	0	0	0	0	0	0	0	0	0	0	0	0	0	0	0	0	0	252	0	533	1563	0	0	0	0	0	0	199	736
0	0	0	0	0	0	0	0	0	0	0	0	0	0	0	0	117	0	0	0	0	0	690	0	0	0	1030	0	0	0	0	0	0
0	0	0	0	0	0	0	0	0	0	0	0	811	0	0	0	0	0	0	0	0	0	0	0	0	0	0	0	0	0	0	0	0
0	0	0	0	0	0	0	0	0	0	0	0	0	0	0	0	0	0	0	0	0	0	0	0	0	0	0	0	0	0	682	0	0
0	0	0	0	0	0	0	0	0	0	0	0	0	0	0	0	0	0	0	279	0	0	0	0	0	0	0	0	0	0	0	0	0
0	0	0	0	0	0	0	0	0	0	0	0	0	0	0	0	0	0	0	0	0	0	0	0	0	0	0	0	0	254	0	0	0
0	0	0	0	0	0	0	0	0	0	0	0	0	406	0	0	0	0	0	0	0	0	0	0	0	0	0	0	0	0	0	0	0
0	0	0	0	0	0	0	0	0	0	0	0	0	0	0	0	0	0	0	0	0	0	0	0	0	226	0	0	0	0	0	0	0
0	0	0	0	0	0	0	0	0	0	0	0	0	0	0	0	0	0	0	294	0	0	0	0	0	0	0	0	0	0	0	0	0
0	0	0	0	0	0	0	0	0	0	0	0	0	0	0	0	0	0	0	0	0	0	0	0	0	0	0	0	0	0	0	0	0
0	0	0	0	0	0	0	0	0	0	0	0	0	0	0	0	0	0	0	0	0	0	0	0	0	0	0	214	0	0	0	0	0
0	172	0	0	0	0	0	0	0	0	0	0	0	0	0	0	0	0	0	0	0	0	0	0	0	0	0	0	0	0	0	0	0
0	0	0	0	0	178	0	0	0	0	0	0	0	0	0	0	0	0	0	0	0	0	0	0	0	0	0	0	0	0	0	0	0
0	0	0	0	0	0	0	0	0	0	0	0	0	0	0	0	0	0	0	0	0	0	0	0	0	0	0	0	0	0	307	0	0
0	0	0	0	0	0	0	0	0	0	0	0	0	0	0	0	75	140	0	0	0	0	0	0	0	0	0	0	0	0	0	0	0
0	0	0	0	0	0	0	0	178	0	0	0	0	0	0	0	0	0	0	0	0	0	0	0	0	0	0	0	0	0	0	0	0
0	0	0	0	0	0	0	0	0	0	0	1462	0	0	0	0	0	0	0	0	0	0	0	0	0	0	0	0	0	0	0	0	0
215	0	0	0	0	0	0	0	0	0	0	0	0	0	0	0	0	0	0	0	0	0	0	0	0	0	0	0	0	0	0	0	0
0	0	0	0	0	0	0	0	0	199	0	0	0	0	0	0	0	0	0	0	0	0	0	0	0	0	0	0	0	0	0	0	0
0	116	0	0	0	0	0	0	0	0	0	0	0	0	0	0	0	0	0	0	0	0	0	0	0	0	0	0	0	0	0	0	0
0	0	0	0	0	0	0	0	0	0	0	0	0	0	0	0	0	184	0	0	0	0	0	0	0	0	0	0	0	0	0	0	0
0	0	0	0	0	0	0	0	0	0	0	0	0	0	0	0	0	0	0	0	0	0	0	56	0	0	0	0	0	0	0	0	0
0	0	0	0	0	0	0	0	0	0	0	0	0	0	0	0	0	0	0	0	0	350	0	0	0	0	0	0	0	0	0	0	0
0	0	0	0	0	0	0	0	0	0	0	0	0	0	0	0	0	0	0	0	0	0	0	0	0	0	0	0	0	157	0	0	0
0	0	0	0	0	0	0	0	0	0	0	0	0	0	0	0	0	0	0	0	0	0	0	0	0	0	0	0	0	0	0	0	0
0	0	0	0	0	0	0	0	0	0	0	0	0	0	0	0	0	0	0	162	0	0	0	0	0	0	0	44	0	0	0	0	0
0	0	0	0	0	0	0	0	0	0	0	0	0	0	0	0	0	0	0	0	0	0	0	0	0	0	198	0	0	0	0	0	0
0	0	0	0	0	0	0	0	0	0	0	0	0	0	0	0	0	0	0	0	0	0	496	0	0	0	0	0	0	0	0	0	0
0	0	0	0	0	0	0	0	0	0	0	0	0	0	0	0	0	0	0	190	0	0	0	0	0	0	0	0	0	0	0	0	0
0	0	0	0	0	0	0	0	0	0	0	678	0	0	0	0	0	0	0	0	0	0	0	0	0	0	0	0	0	0	0	0	0
0	0	0	0	0	0	0	0	0	0	0	0	0	0	0	0	0	0	0	0	0	0	0	0	0	0	0	0	0	0	0	0	0
1136	1708	1365	456	137	455	284	476	178	947	748	17211	21231	14517	6298	374	19218	16533	11000	15844	18327	17329	13604	21085	25691	34413	24038	14847	18187	35657	18103	16103	10183

Table S4. Calibrated ^{14}C ages of organic materials from the sediment core 21SJ.

core depth (cm)	material	conventional ^{14}C age (BP ± 1σ)	calibrated age (BP)	
			(BP)	(CE/BCE)
**229.7**	terrestrial plant (leaf)	694 ± 35	654	**1296 CE**
**250.5**	terrestrial plant (twig)	860 ± 30	758	**1192 CE**
**266.5**	terrestrial plant (leaf and seed)	1060 ± 30	958	**992 CE**
**295.5**	terrestrial plant (leaf)	1290 ± 30	1226	**724 CE**
**359.7**	terrestrial plant (leaf)	2250 ± 30	2229	**279 BCE**
